# Count every newborn: EN-BIRTH study improving facility-based coverage and quality measurement in routine information systems

**DOI:** 10.1186/s12884-020-03427-4

**Published:** 2021-03-26

**Authors:** Allisyn C. Moran, Jennifer Requejo

**Affiliations:** 1 Department of Maternal, Newborn, Child and Adolescent Health and Ageing, World Health Organization, Geneva, Switzerland; 2 Division of Data, Analysis, Planning and Monitoring, United Nations Children’s Fund, Headquarters, New York, NY USA

## Why was the EN-BIRTH study needed?

Unacceptably, 2.4 million newborns were estimated to have died during their first 28 days in 2019 [1]. Additionally at least 2 million babies each year were stillborn in the last 3 months of pregnancy [2], many during labour and many to the 0.3 million mothers who die from maternal causes each year [3]. Millions more babies were born too soon, at increased risk of long-term disabilities [4]. In 2014, the *Every Newborn* Action Plan (ENAP) [5, 6] was endorsed by 194 Member States, including a commitment to end preventable newborn deaths and stillbirths. The first ever global target for neonatal mortality reduction was included in Sustainable Development Goal (SDG) 3 [7]. To attain universal health coverage and meet SDG 3 by 2030, countries need to scale up evidence-based interventions, including for newborn health. Hence timely and high-quality data on outcomes and coverage are crucial, especially through national health information systems. During pandemics, stillbirths and neonatal deaths may be increased [8], further underlining the need for data through routine systems.

Core indicators to track progress in maternal and newborn health were prioritized through evidence review, and an inclusive consultation process undertaken through ENAP [9, 10]. In high burden settings, the majority of comparable data for these indicators are currently collected through population-based surveys, and no rigorous validation studies have been undertaken until now for facility-based maternal and newborn indicators in routine health information systems [10]. A multi-partner measurement improvement roadmap [11] was developed for 2015–2020 to improve the ENAP core indicator definitions and to test their measurement validity – including capturing care for newborns at risk or with complications – and inform feasibility of measurement. This roadmap highlighted a major gap for measurement of coverage and quality of care, including service readiness.

**Fig. 1:**
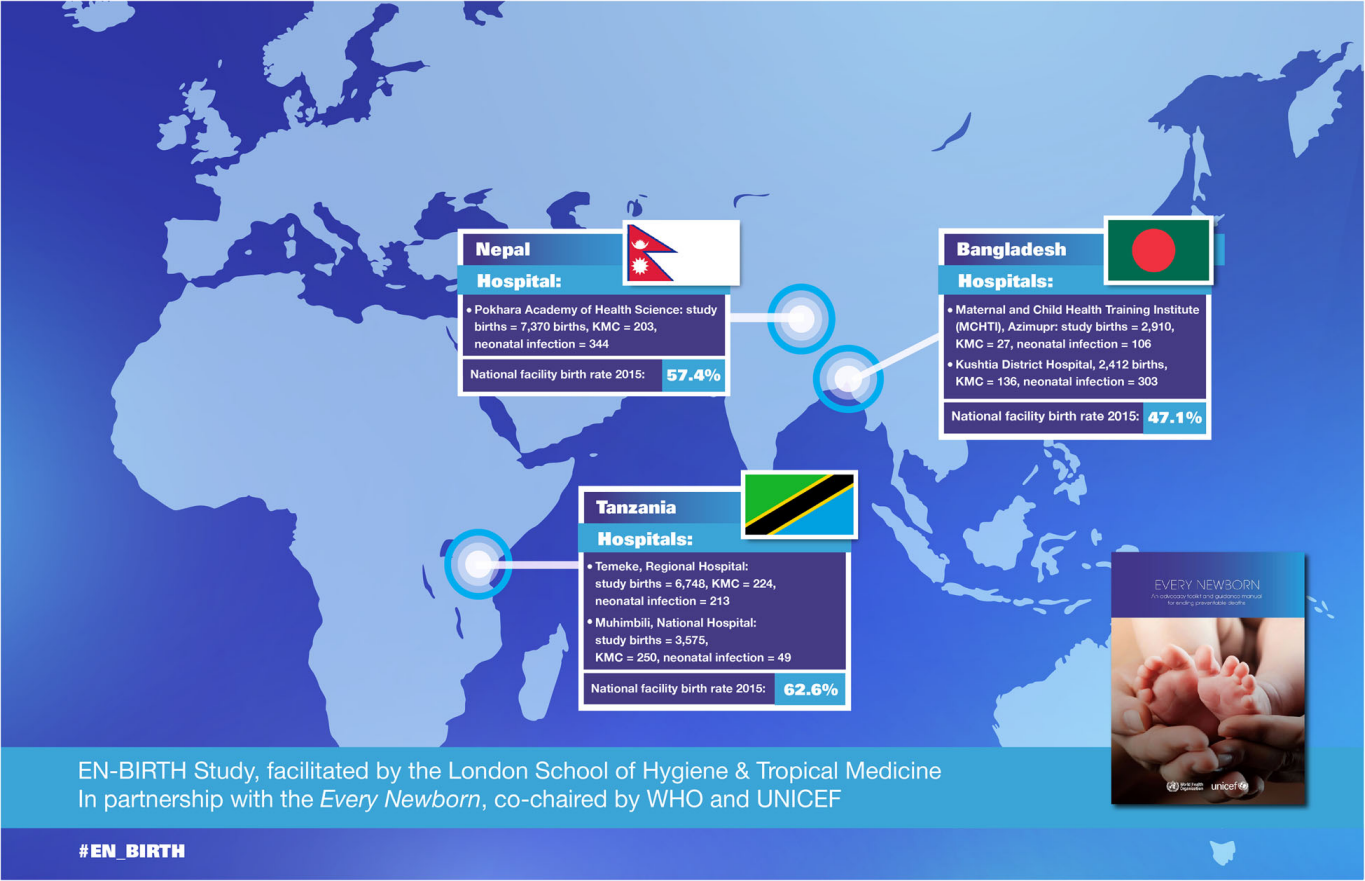
EN-BIRTH five study sites in three countries. National facility birth rates are for 2013-2018 [13]

The *Every Newborn* - Birth Indicators Research Tracking in Hospitals (EN-BIRTH) study is directly linked to the ENAP measurement improvement roadmap, and ultimately SDG3. The study’s primary aim was to validate, by comparison to direct clinical observation as the gold standard, data from routine facility registers and women’s survey report for capturing facility-based coverage and quality of care [12]. EN-BIRTH was conducted in five hospitals providing comprehensive emergency obstetric and newborn care in three high-burden mortality countries: Tanzania, Bangladesh, and Nepal (Fig. 1). The multi-country EN-BIRTH team observed 23,471 births and 840 kangaroo mother care (KMC) mother-baby pairs, in addition to collecting information on 1015 admissions for neonatal infection. The three country research teams represent ENAP priority countries from sub-Saharan Africa and south Asia. The multi-country research team actively co-designed the study, facilitated by a team at the London School of Hygiene & Tropical Medicine (LSHTM) and funded by Children’s Investment Fund Foundation (CIFF). The large quantitative dataset and standardised approach to qualitative data collection enabled the synthesis of barriers/enablers to collection and use of data in routine registers.

## What is new and what have we learned?

**Fig. 2:**
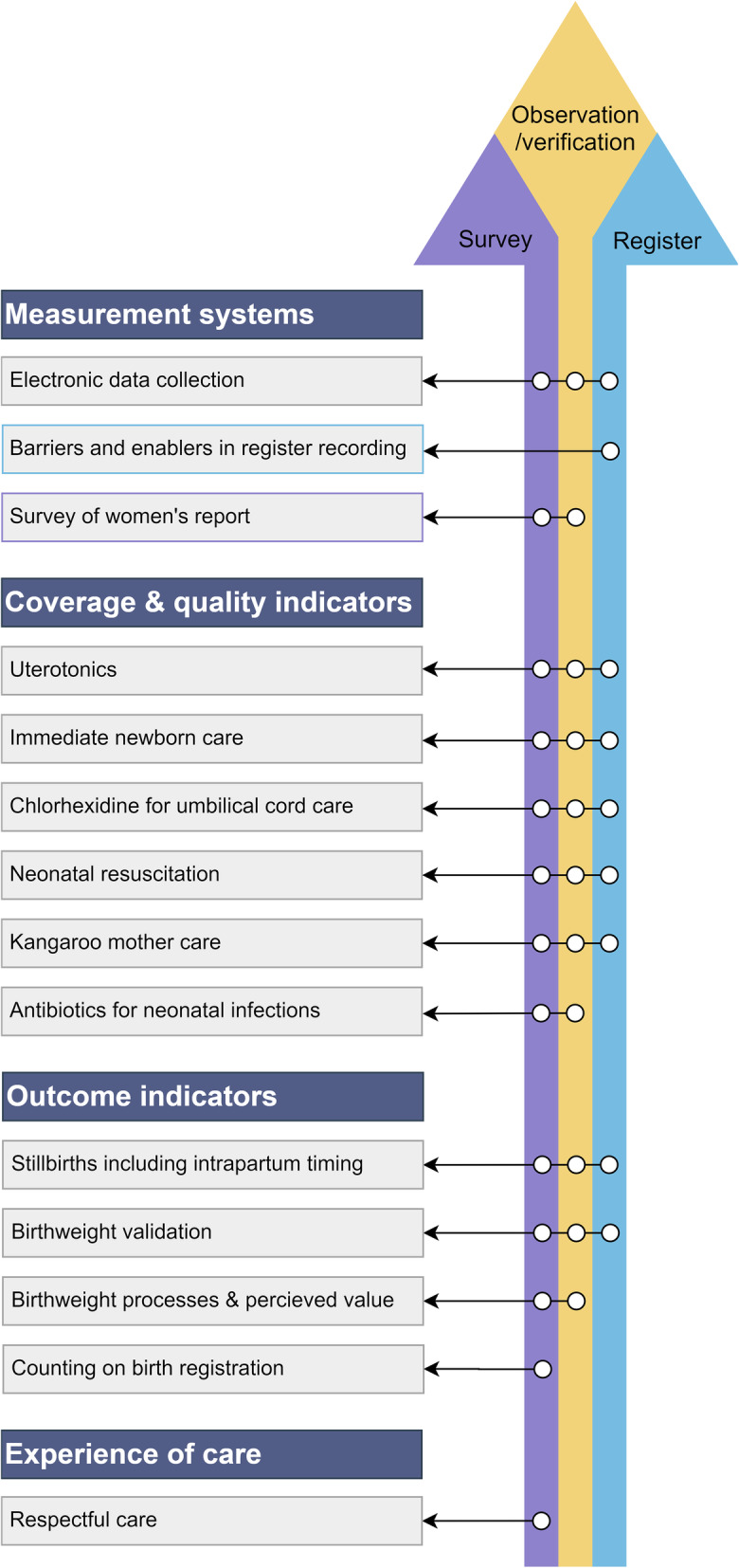
Overview of the three data types in EN-BIRTH study and the 14 papers in the supplement. Key findings for supplement papers are presented in Additional file 1

The EN-BIRTH study provides many important findings to advance measurement to drive change. Here we focus on the 14 papers in this supplement (Fig. 2). The overall validation results from EN-BIRTH are published separately [14]. This supplement starts with three papers on measurement systems covering: EN-BIRTH electronic data collection [15], survey-report for validity of 33 indicators [16], and barriers/enablers to recording in routine registers [17]. Subsequent papers detail findings for the following maternal and newborn coverage indicators: uterotonics to prevent postpartum haemorrhage [18], immediate newborn care including breastfeeding practices [19], chlorhexidine for umbilical cord care [20], neonatal resuscitation [21], KMC [22], neonatal infection antibiotic management [23]. Two papers assess validity and data quality for the outcomes of birthweight [24] and stillbirth [25]. Measurement of respectful maternal and newborn care is assessed in one site (Nepal) [26]. Processes and perceptions for birthweight [27] and birth registration measurement [28] are examined in the Tanzanian sites. These papers all outline actions for improving measurement now and proposing what research is needed next.

Women’s survey-report performed fairly well for birthweight, although with more heaping than in register data [24]. EN-BIRTH shows for the first time that such surveys may be a useful tool for capturing information on contact indicators, such as admission to a neonatal unit [23] or KMC ward. However, survey questions have low accuracy for most maternal and newborn clinical interventions. For example, the results on measuring either the numerator or denominator for antibiotic use for neonatal infections are consistent with findings regarding low survey validity for antibiotic treatment for childhood pneumonia [29]. Clinical interventions that include timing pose additional challenges in survey data collection platforms. For instance, early initiation of breastfeeding within 1 h of birth was overestimated by women's survey report, compared to time-stamped observer data [19]. More research is needed on the validity of survey questions for clinical interventions, including less focus on a rigid time schedule.

Routine hospital registers had high validity for most clinical interventions assessed in at least one hospital, provided the register design included the indicator. Registers performed particularly well for indicators regarding uterotonics for prevention of postpartum hemorrhage, chlorhexidine cord application, and have potential for neonatal bag-and-mask resuscitation [18, 20, 21]. Design of registers is critical for improving reporting accuracy, even the labour ward register designs varied across the three countries. There is much work to be done to standardise register content and link registers with individual patient case notes to reduce the number of times health workers are required to record duplicate data. To increase the data availability at the national level, registers will first need to be standarised to capture a parsimonious indicator list, including the linked data elements for numerator and denominator of each indicator. Second, these data need to be linked with other hospital documentation, including the flow into digital platforms. Implementation of the standardised registers need local ownership, to increase the likelihood of uptake and, importantly, local use of routine data in improving care and monitoring.

Quality of care had many gaps compared to global standards, notably regarding timing of care. For example, although provision of uterotonics to prevent postpartum hemorrhage was universally high across all five hospitals, the observational data showed that overall only 16% of women received uterotonics within 1 min after birth [18]. Regarding neonatal resuscitation, most non-breathing newborns were observed to receive bag-mask-ventilation, but overall only 1% within the recommended 1 min after birth [21]. Whilst nearly all babies were weighed within 1 h of birth (97%), only 16% were weighed using digital scales [24]. Most stillbirths were weighed, apart from one site [25]. Antibiotic stewardship was also a serious issue across the study sites. Overall only 11% of newborn inpatients had a blood culture; even fewer had a lumbar puncture (< 1%) and few newborns received the recommended antibiotics for the optimum duration. For KMC, ward registers accurately captured admission to care (a service contact measure), yet there were gaps identified in quality of care, especially duration and feeding support [22]. These findings indicate a need for further research to determine the underlying causes of the poor quality of care so that remedial action can be taken.

A novel finding, based on the large number of women who had a caesarean section (6698) in this study, is the effect of mode of birth on other care practices and register or survey report measurement. Given the rising global rates of caesarean sections, care practices and measurement implications require more study.

## What next for improving and using data on coverage and quality?

EN-BIRTH is the first multi-site, facility-based study validating measures in routine register data for maternal and newborn care and in women’s survey-report for newborns with complications. Both data sources have value, yet both have limitations. Women’s survey-report can be used effectively for collecting certain information, notably service contact points – as is already done for antenatal care, institutional birth, and postnatal care. EN-BIRTH results show high validity for survey questions on admission both to KMC ward and newborn care ward. These questions performed well, but further testing is required among those whose newborns were not admitted; longer recall (2-5 years) and survey sample size also need to be considered [16]. EN-BIRTH clearly adds to findings from previous research that surveys are not an appropriate tool for capturing valid information on clinical interventions provided around the time of birth, and that more work is needed to refine survey indicators based on timing of care, such as early initiation of breastfeeding [30, 31]. Surveys may be useful for measuring experience of care, but there are notable challenges in women’s ability to report their experience of care, especially when asking questions in or near the facility [26, 32].

Registers in labour wards, operating theatres, KMC wards and newborn care wards have tremendous potential to track facility-based maternal and newborn interventions, maternal and newborn outcomes, and stillbirths. Implementation research is needed to design registers to include necessary data elements and to optimise register filling, local use, and data flow, including linkage to electronic platforms already used in low- and middle-income countries (LMICs) [33]. Capturing detailed aspects for quality of care is not likely to be feasible in routine registers, and it requires specific linked datasets (e.g. on neonatal care wards). Timing components such as early initiation of breastfeeding, uterotonic administration, and resuscitation are challenging to record and may need special studies. More research is required on ways to capture and improve the delays in service delivery found in this study, since such delays can cause deaths.

The ENAP measurement improvement roadmap published in 2015 was instrumental in bringing together a wide team and undertaking the important yet challenging EN-BIRTH study. The World Health Organization (WHO) and United Nations Children's Fund (UNICEF) with ENAP partners are reviewing these findings, alongside other evidence, to update recommendations on newborn indicators, including on the metadata (i.e. definition, numerators/denominators and recommended data collection platforms). This work is crucial given the launch of new ENAP coverage targets for all countries from 2020 to 2025, including a novel target for small and sick newborn care. In addition, the ENAP measurement improvement roadmap will be updated to set out clear priorities for research in the next 5 years.

As well as being an ambitious research study, the EN-BIRTH team is an example of an equitable partnership, with built-in opportunities for multi-directional learning across study sites. At least four linked PhDs are being undertaken by researchers participating in the study. Given commitments to decolonising global health, institutions and funders should support other studies that build capacity of in-country teams for leadership and analytical skills. More data alone will not change outcomes – we need to foster the next generation of leaders and researchers to improve the data, and to use data in the highest burden settings.

EN-BIRTH study shows that a large increase in data on maternal and newborn health could be achieved by strengthening routine health information systems, enabling improved clinical care, and better tracking towards the ENAP coverage targets and ultimately the SDGs. With the right actions in the next few years, we can improve data and most importantly increase coverage, equity, and quality of care to save the lives of every mother and every newborn, everywhere.

## Supplementary Information

**Additional file 1.** Key findings of EN-BIRTH study.

## Data Availability

The datasets generated during and/or analysed during the current study are available on LSHTM Data Compass repository, https://datacompass.lshtm.ac.uk/955/.

## Abbreviations

CIFF: Children’s Investment Fund Foundation
ENAP: *Every Newborn* Action Plan
EN-BIRTH: *Every Newborn*-Birth Indicators Research Tracking in Hospitals study
icddr,b: International Centre for Diarrhoeal Disease Research, Bangladesh
IHI: Ifakara Health Institute
LMIC: low- and middle-income countries
KMC: kangaroo mother care
LSHTM: London School of Hygiene & Tropical Medicine
MUHAS: Muhimbili University of Health and Allied Sciences
SDG: Sustainable Development Goal
UNICEF: United Nations International Children’s Emergency Fund
WHO: World Health Organization
